# Rural Community as Context and Teacher for Health Professions Education

**DOI:** 10.7759/cureus.866

**Published:** 2016-11-07

**Authors:** Kedar Baral, Jill Allison, Shambu Upadhyay, Shital Bhandary, Shrijana Shrestha, Tia Renouf

**Affiliations:** 1 Department of Community Health Sciences, Patan Academy of Health Sciences; 2 Medicine, Memorial University of Newfoundland; 3 Dean of Medicine, Patan Academy of Health Sciences; 4 Emergency Medicine, Memorial University of Newfoundland

**Keywords:** nepal, rural medicine

## Abstract

Nepal is a low-income, landlocked country located on the Indian subcontinent between China and India. The challenge of finding human resources for rural community health care settings is not unique to Nepal. In spite of the challenges, the health sector has made significant improvement in national health indices over the past half century. However, in terms of access to and quality of health services and impact, there remains a gross urban-rural disparity. The Patan Academy of Health Sciences (PAHS) has adopted a community-based education model, termed “community based learning and education" (CBLE), as one of the principal strategies and pedagogic methods. This method is linked to the PAHS mission of improving rural health in Nepal by training medical students through real-life experience in rural areas and developing a positive attitude among its graduates towards working in rural areas.

This article outlines the PAHS approach of ruralizing the academy, which aligns with the concept of community engagement in health professional education. We describe how PAHS has embedded medical education in rural community settings, encouraging the learning context to be rural, fostering opportunities for community and peripheral health workers to participate in teaching-learning as well as evaluation of medical students, and involving community people in curriculum design and implementation.

## Introduction and background

Nepal is a low-income, landlocked country located on the Indian subcontinent between China and India. With a population of 30 million, nearly 83% live in rural areas that are mainly hills and mountain regions. Per capita Gross Domestic Product (GDP) and per capita Gross National Income (GNI) are projected to be US$703 and 717, respectively, and about one-fourth of the population (25.16%) lives below poverty lines [1]. Health expenditure per capita remains low at US$18.09, out of which 55% is borne by households (out-of-pocket) at the time of service [2].

The challenge of finding human resources for rural community health care settings is not unique to Nepal. However, the small country has been innovative in its attempts at overcoming primary health care challenges. The implementation of a network of Female Community Health Volunteers is one of the examples; these volunteers serve as a conduit for primary health information, support and care in a system that also utilizes task shifting and upskilling to fill health care needs [3]. As a result, Nepal’s health sector has made significant improvements in national health indices over the past half century [4]. However, in terms of access to and quality of health services and impact, there remains a gross urban-rural disparity. For example, while the national average life expectancy is about 67 years, it is about 10 years lower for the remote rural population [5], and the infant mortality rate in the rural mountainous areas is more than double that of urban areas [6]. 

One of the reasons for such disparities is a lack of quality health services in rural and remote areas partly related to ineffective medical education, inefficient deployment and poor retention of health human resources there. Two-thirds of the estimated 4,000 physicians engaged in the health sector are concentrated in urban areas [5], while rural health care facilities remain understaffed. Past attempts at bridging the gap have included the development of the Medical Doctorate in General Practice (MDGP) program in the 1980s as collaboration between the Institute of Medicine at Tribhuvan University in Nepal and the University of Calgary with support from the Canadian government [7]. This program is based on a curriculum to train qualified physicians to be competent generalists with skills suited to rural and district hospital practice. The curriculum and model have been adopted by a number of countries including India [8]. However, this program has remained under-recognized due to a lack of career development opportunities within the national health system (NHS) and produces only a few graduates per year.

As in most countries, health professions education in Nepal is predominantly urban centered and generally focused on the cure of individuals whose problems represent only the tip of the iceberg of the prevailing community health problems [9-10]. As a result, health professions education programs have not been very successful in helping graduates understand the relevance of community and societal needs or in preparing them adequately to meet such needs [10]. Furthermore, the majority of medical schools in Nepal have not embraced the social accountability principle that directs medical schools to partner and collaborate with communities, governments, healthcare organizations and health professionals for improved health outcomes in the communities they serve [11-12].

## Review

It is in this context that, PAHS was established in 2008 with a considerably different mission, which is directed by the social accountability principle [13] to help reduce existing rural-urban health inequity in Nepal.

*“PAHS is dedicated to sustained improvement of the health of the people in Nepal, especially those who are poor and living in rural areas, through innovation, equity, excellence and love in education, service and research.” - *Patan Academy of Health Sciences mission statement

In order to achieve its stated mission of improving rural health in Nepal by training medical students and other health workers, there is a need for real-life experience in rural areas and development of a positive attitude among graduates of working in rural areas. PAHS has, therefore, adopted CBLE as one of the principal strategies and pedagogic methods. This is one of several measures it has taken for ruralizing the academy and, thereby, meeting the challenge of producing physicians who are able to work in underserved areas in the future [14-18].

First launched as a program in June 2010, the PAHS undergraduate medical education curriculum emphasizes the importance of population health issues, a sound understanding of real-life context of rural communities and the NHS. The community health sciences (CHS) course was recognized as a major component of the curriculum allocating 25% of total curricular time [8]. PAHS’s CBLE program employs a setting-based approach [9] exposing students for varying durations to diverse contexts that include urban slums, rural communities and different levels of rural health care institutions [14] within the NHS. Such experiential learning opportunities are expected to help graduates obtain a firm grasp of concepts and principles of preventive health and social determinants of health while developing the necessary skills in management, epidemiology and research. Students get firsthand opportunities to hear from the community about their needs and also learn about community strengths for undertaking the actions required for health improvement.

**Figure 1:**
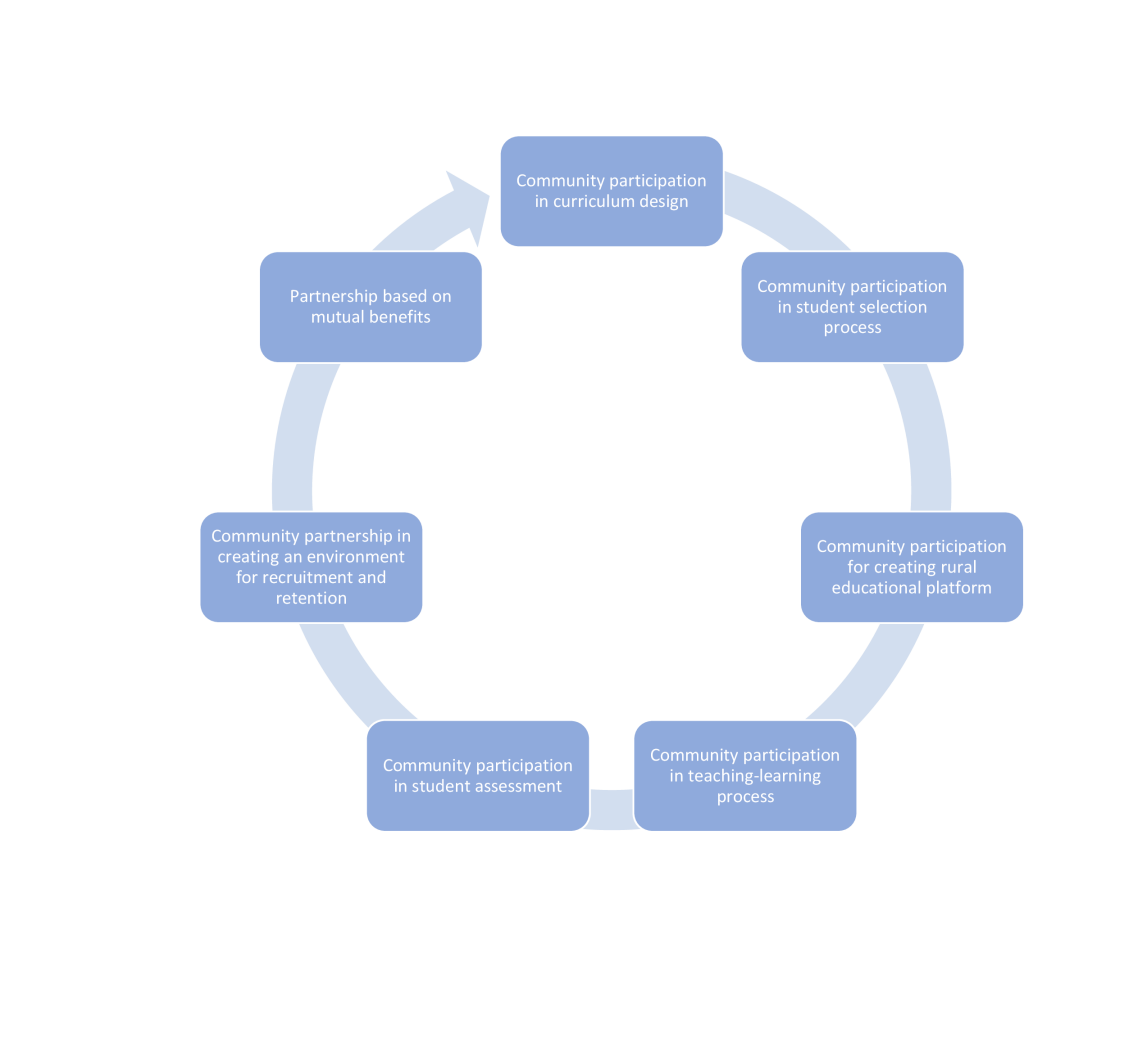
Community engagement for ruralizing PAHS program

The PAHS approach of ruralizing the academy aligns with the concept of community engagement [19] in health professional education by embracing the opportunity for education to be embedded in rural community settings, encouraging the learning context to be rural, fostering opportunities for community and peripheral health workers to participate in teaching-learning as well as evaluation of medical students, and involving community people in curriculum design and implementation. The CBLE strategy adopted by PAHS continues to engage students, faculty and the academy itself with rural communities, local government bodies, community based organizations (CBOs) as well as the NHS. PAHS is also planning a school of public health and a nursing school based on the same principles. Figure 1 below depicts various approaches PAHS has initiated for ruralizing the academy.

### Community participation in curriculum design

The PAHS undergraduate medical curriculum development process underwent a wide consultation with stakeholders including rural communities and consumer groups. The community specifically contributed in defining the mission and the graduate attributes [20] that guided the further steps of curriculum development [17]. Community participation thus underpins all aspects of the partnership ensuring that the needs of the community inform the curriculum in a dynamic process that reflects the values PAHS aspires to instill in its graduates [14]. 

### Community participation in student selection process

Rural engagement begins very early at PAHS, starting with the student selection process that gives preferential credits to those who are from rural, remote and disadvantaged sectors of society [8] but have reasonably sound cognitive ability. In order to accomplish this, rural community leaders from prospective teaching districts/areas and leaders from consumers’ society were invited to a workshop aimed at collecting their input in defining the ideal attributes of PAHS graduates. Members of the community are invited to PAHS to be a part of the student selection process and team, especially in assessing candidates’ communication skills as well as sensitivity, compassion and empathy towards societal contexts and societal needs. Selected rural community representatives with a role in developing their own communities are provided logistic support by PAHS to come and participate in the school-entry orientation program of students. They participate as full team members giving motivational speeches and inspiring students with stories of their own work in uplifting their communities. At the outset of the students’ journeys into this field, these community role models share their experiences as change agents as well as their insights on what communities expect from future medical professionals [14-15].

### Community participation for creating rural educational platforms

PAHS has developed partnerships with women’s groups, women’s cooperative groups, CBOs, Red Cross, local community leaders as well as local health institutions at the community level. Agreements have been made with Ministry of Health and Population (MOHP) for mobilizing health sector collaboration at various levels for CBLE field postings of students. In addition, support for program implementation has been gathered through intersectoral collaboration with local administrative bodies of the government such as District Development Committees, Village Development Committees (VDCs), police and security units as well as nongovernmental organizations (NGOs) working in the field of social mobilization and community development.

### Community participation in teaching and learning process

PAHS has adopted CBLE both as the experiential strategy for preparing medical graduates for work in rural communities and as a pedagogical approach. It locates medical education in community context and helps infiltrate the academic process with a solid understanding of rural health needs. The community thus serves as a platform for both teaching and learning activities. Community members participate in planning and implementation of postings, and they are also actively involved in the teaching-learning process by providing and demonstrating local context. They are involved in all levels of discussion. The community partnership also ensures that students: have safe, adequate and appropriate lodging and meals; and are supervised, guided and mentored in day-to-day activities during the posting period. Community members are also key informants and provide deeper insights into the rural life’s opportunities, challenges and community assets that are important for health professionals. Finally, they provide constructive feedback to the students on their presentation of key findings during the exit meeting held at the end of each posting.

There are seven postings spread over five years in the curriculum with duration beginning with one week and gradually increasing to six months as students advance through the program. These postings provide exposure to diverse rural communities and all tiers of the NHS. Each of these seven CBLE postings consists of a set of generic as well as specific technical learning objectives. The generic objectives include observing the settlements, drawing social maps, describing demographic/socioeconomic conditions, describing sociocultural practices relating to health, identifying the roles of organizations involved in social development including health and describing functions of (local) Health Facility Management Committees (HFMCs). Students also interact with the stakeholders, including patients, to explore opportunities and challenges of the posting areas, participate in day-to-day activities of the health facility and make suggestions and recommendations to the stakeholders for improvement of services. The posting-specific objectives differ for each posting and are tailored to the community circumstances and needs based on consultation at the community level [14].

### Community participation in student assessment

PAHS has developed an innovative blend of both content (cognitive) and process (non-cognitive skills and behaviors) evaluation of learning with both formative and summative measures. It is in the area of skills and behaviors that the community is directly involved in assessing the students’ day-to-day activities, behavior, attitude, communication, empathy, compassion and relationship-building shown during their stay in the community. Community members (including heads of households hosting students during their field stay), local community leaders and local health workers are involved in assessing students using objectively structured formats developed for this specific purpose. The assessment of students by community partners contributes to formative evaluations but is also taken into account by faculty supervisors in summative evaluations. This is a distinct feature of PAHS as an academic institution wherein the partnership with the community extends to the opportunity and responsibility for lay/community members to contribute to the summative evaluation of its students.

### Community partnership in creating enabling environment for recruitment and retention

PAHS is expanding partnerships with the government and local bodies in order to create enabling environments for the deployment and retention of graduates in rural areas. Such enabling environments include a supportive and consistent system of postings to rural sites and investing in medical students' education. PAHS has established a collaborative scholarship scheme where communities and local governments pay for students’ education based on a commitment to return of service locally. At the same time, PAHS faculty advocate for systemic changes that will ensure graduates can return to their home communities to fulfill both need and obligation [14, 16-17].

### Partnership based on mutual benefits

Partnerships with communities are developed on the basis of mutual benefits. While the community serves as both a learning platform and resource for the academy, it also receives a range of benefits during this collaborative process. Local needs are taken into consideration while developing specific objectives and detailed activities of student field postings. In the field, students assess the demographic and health status of an assigned catchment population by applying qualitative and quantitative methods learned in school, analyzing primary and secondary data, and preparing reports. Such reports help in program planning for catchment communities. Students carry out health facility assessment surveys, identify low-performing program areas and share their findings with service providers and program managers to organize discussion for drawing feasible suggestions or action plans for improvement.

Community members and local health workers who are directly involved in guiding and supervising students are given status as facilitators from PAHS for each field posting. There are different levels of posting of various duration; as the community member might be different each time, multiple members have received facilitator status. Those who are in the health system and qualify for PAHS faculty status are recognized as such based on eligibility criteria. This recognition is key to ruralizing the academy, designating teachers across a wide range of skills and attributes who come from different geographical locations.

Additionally, the community benefits from increased access to tertiary care through a referral mechanism worked out in partnership with local health workers and institutions. Patan Hospital, the principal teaching hospital of PAHS, is a tertiary referral center. If a health worker refers a patient from a teaching and learning site, that patient will not be put in the queue for registration but will see the appropriate specialist directly. Students are deeply involved in health promotion while they are in the community and assist local health facility and staff in day-to-day service, depending on posting level and student skill level.

During their community posting, advanced-year students are directly involved in providing basic primary health care to communities while they are supervised by local health workers. Additional rewards come from students and community members keeping in touch as students are promoted in their studies. Moreover, students become involved in advocacy activities, both locally and at the policy level, as they come to understand and argue for programs that will benefit the community [14]. Efforts are being made to expand this partnership to open up new avenues for collaborative projects and programs like community-based implementation research, quality improvement programs and strengthening of local systems.

Although aware that it may come in the future, PAHS has not noted any obvious community fatigue. In order to prevent fatigue, the school works closely with district-level officials to rotate the VDCs that are chosen for postings. Efforts are made to ensure that each VDC has the capacity to host the students, make use of information gathered and support the projects in which students engage while in the community. 

## Conclusions

As graduates from PAHS move forward into careers as physicians, one key marker of the success of PAHS in advocating for rural primary health care will be the willingness of government to post graduates in the communities they have pledged to serve. Ongoing evaluation of graduate placement locations and systemic support, graduate satisfaction with the postings and level of comfort in providing services will provide important data for both advocacy work and curriculum development. Another important marker for success will be the satisfaction of communities who have invested in the development of the program and provided educational support and opportunities. Success will be realized when local community leaders and members of mothers’ groups, auxiliary health care providers and community members express their satisfaction that health care is being delivered with their needs in mind.

So far, PAHS has engaged communities with many successes and no major associated problems. Rather students, faculty and the academy are receiving continued support and enthusiastic involvement from communities who feel proud to be part of the program. There are positive changes in health-seeking behaviors and level and quality of care. The various approaches of ruralizing the academy described in this article are in alignment with, as well as suggestive of, literature findings [10, 15, 21-25], particularly in improving overall coverage of rural areas. Therefore, adoption of such approaches is expected to help reduce the existing rural-urban health disparity in Nepal, which is the overall vision of PAHS as an institution. While it is premature to draw definitive conclusions until PAHS graduates enter the rural health workforce and the effectiveness of the program is evaluated, the positive aspects of community engagement are already a success story.
